# Successful management of metachronous dual primary esophageal carcinomas: first report of consecutive adenoid cystic carcinoma and squamous cell carcinoma with exceptional treatment outcome—a case report

**DOI:** 10.3389/fonc.2026.1760081

**Published:** 2026-05-22

**Authors:** Yuxi Bai, Lidan Geng, Xiyue Yang, Qiuling Shi, Jie Li, Gang Feng

**Affiliations:** 1 Department of Oncology, Mianyang Central Hospital, School of Medicine, University of Electronic Science and Technology, Mianyang, Sichuan, China; 2 Day-Treatment Center, Mianyang Central Hospital, School of Medicine, University of Electronic Science and Technology, Mianyang, Sichuan, China; 3 State Key Laboratory of Ultrasound in Medicine and Engineering, School of Public Health and Management, Chongqing Medical University, Chongqing, China

**Keywords:** adenoid cystic carcinoma, case report, esophageal neoplasms, multiple primary malignancies, radiotherapy, second primary malignancy

## Abstract

**Background:**

Esophageal adenoid cystic carcinoma (ACC) is an exceptionally rare malignancy, and its association with second primary malignancies (SPMs) remains poorly characterized. This report presents the first documented case of metachronous esophageal squamous cell carcinoma (SCC) developing outside the radiation field following curative treatment of esophageal ACC.

**Case presentation:**

A 58-year-old male with a substantial smoking history presented with dysphagia in 2022. Diagnostic evaluation confirmed primary esophageal ACC (pT2N0M0, Stage IIA), exhibiting perineural invasion and a high proliferative index (Ki-67 80%). The patient underwent surgical resection followed by adjuvant radiotherapy (50 Gy/25 fractions) to the tumor bed. Postoperative radiotherapy was well tolerated, with only Grade 1 esophagitis and fatigue observed. Sixteen months later, during routine surveillance, a new cervical esophageal lesion was detected 5 cm proximal to the previous anastomotic site, well beyond the prior radiation field. Histopathological examination identified poorly differentiated SCC with distinct immunohistochemical characteristics (P40 diffuse+, Ki-67 30%). Complete remission was achieved following definitive chemoradiotherapy (60 Gy/28 fractions to PGTV, 54 Gy/28 fractions to PTV with concurrent S-1). At the most recent follow-up in March 2026, the patient remained disease-free.

**Conclusion:**

This case illustrates that SPMs may arise outside irradiated regions after ACC treatment, excluding radiation-induced carcinogenesis. The findings emphasize the necessity of comprehensive endoscopic surveillance of the entire esophagus for early detection of secondary primaries in patients treated for esophageal ACC. Furthermore, the favorable therapeutic response demonstrates that curative-intent management remains feasible for metachronous malignancies when identified at an early stage.

## 1 Introduction

Esophageal adenoid cystic carcinoma (ACC) is exceptionally rare, representing only 0.04%–0.16% of esophageal malignancies (1, 2). It exhibits distinct clinicopathological features including aggressive behavior with submucosal infiltration and perineural invasion (3–5). Multiple primary malignancies occur in 5.8%–30.0% of esophageal cancer patients, with synchronous and metachronous tumors comprising 16% and 14% respectively (6–8). Pathogenetic mechanisms involve carcinogen exposure and ‘field cancerization’ of genetically altered mucosal fields, particularly for squamous cell carcinomas (7, 9–12). Although radiotherapy increases second primary malignancy risk after >10 years (13–16), multimodal approaches remain essential for dual primaries (17–21). We previously documented a primary esophageal ACC treated with surgery and adjuvant radiotherapy (5). The patient subsequently developed metachronous squamous cell carcinoma outside the radiation field, which was successfully managed with definitive chemoradiotherapy. To our knowledge, this represents the first reported case of metachronous esophageal ACC and SCC in which radiotherapy was employed as a component of curative-intent treatment for both malignancies, achieving disease-free survival at the most recent follow-up.

## 2 Case presentation

### 2.1 First primary: esophageal ACC (2022–2023)

**Figure 1:**
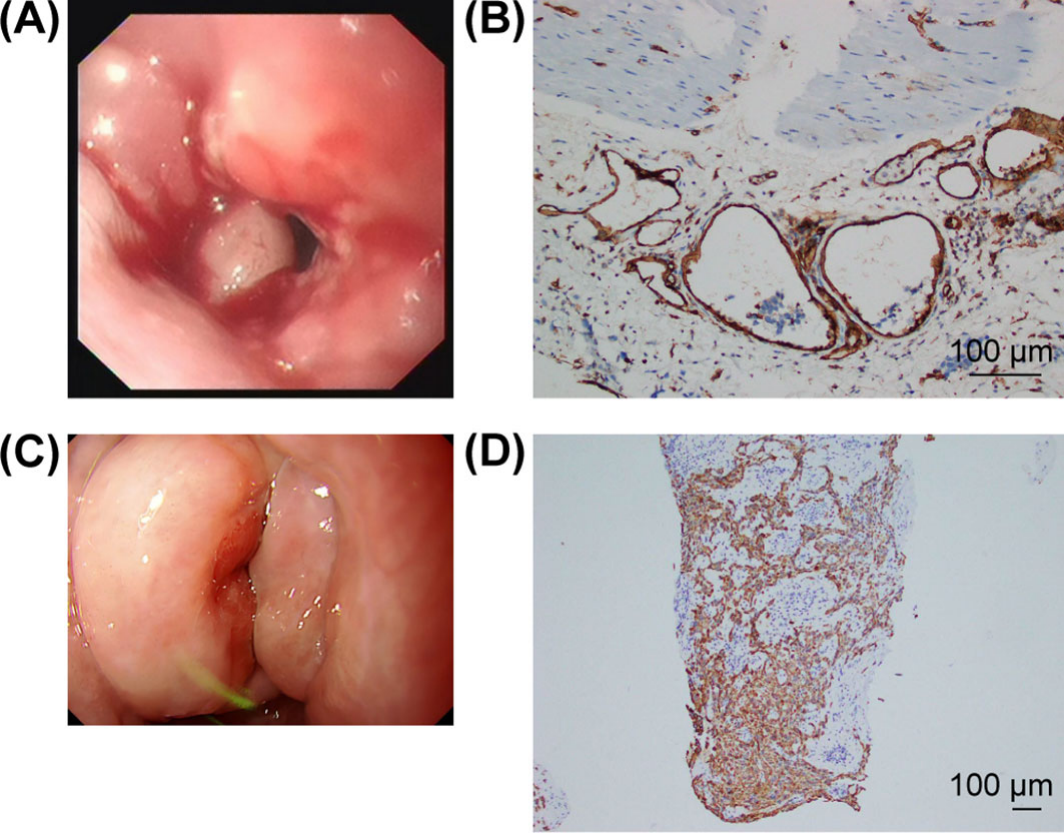
Endoscopic and immunohistochemical findings of metachronous dual primary esophageal carcinomas. **(A)** Initial endoscopic view of lower esophageal ACC. **(B)** CD31 staining of ACC showing vascular invasion with tumor emboli. **(C)** Endoscopic view of cervical esophageal SCC. **(D)** CK5/6 staining confirming SCC.

A 58-year-old male with a 30-pack-year smoking history presented with progressive dysphagia in November 2022. Endoscopy revealed a mass in the lower esophagus (**Figure 1A**). Biopsy demonstrated atypical epithelial cells with a cribriform architecture. In January 2023, the patient underwent thoracoscopic–laparoscopic esophagectomy with gastric conduit reconstruction and left cervical anastomosis.

#### 2.1.1 Pathology

Tumor size: 3.5 × 2.8 cm.

Depth of invasion: Into the adventitia.

Final diagnosis: ACC, pT2N0M0 (AJCC 8th edition, Stage IIA).

Margins: R0 resection (proximal margin 4 cm).

IHC: PCK(+), TTF-1 (–), P40 (focal+), CK5/6 (focal+), CD31+ (vascular invasion with tumor emboli), Ki-67 (80%+) (**Figure 1B**).

Block 15: CD31+ (vascular invasion without emboli).

#### 2.1.2 Adjuvant therapy

**Figure 2:**
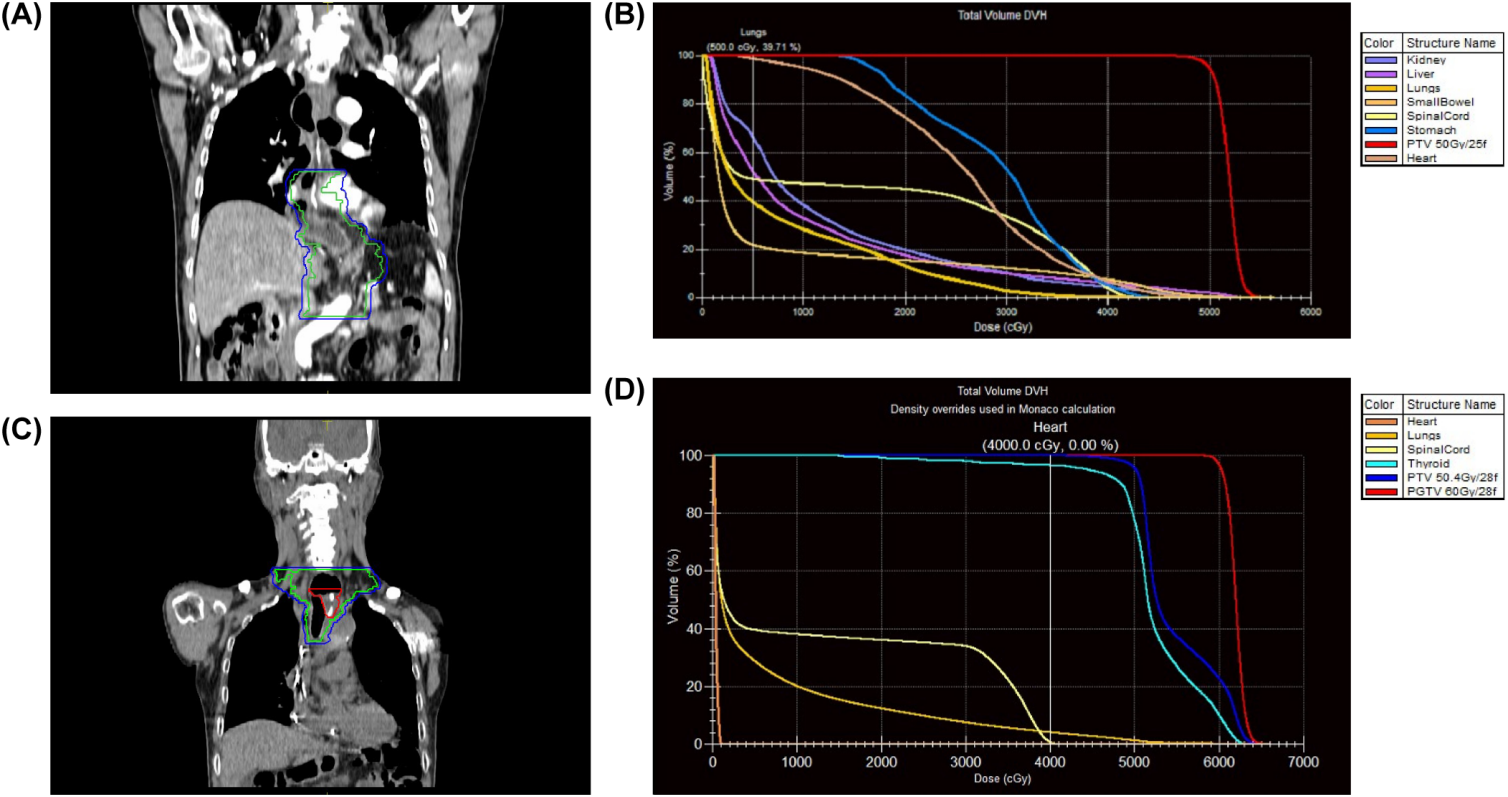
Radiotherapy plans and dose–volume histograms for the two primary malignancies. **(A, B)** IMRT plan and DVH for ACC (50 Gy/25 fractions). For the first radiotherapy course, the mean lung dose was 7.9 Gy, and the mean heart dose was 25.9 Gy. **(C, D)** IMRT plan and DVH for cervical SCC (60 Gy/28 fractions to PGTV, 54 Gy/28 fractions to PTV). For the second radiotherapy course, the mean lung dose was 6.8 Gy, and the mean heart dose was 0.4 Gy. The second lesion was located entirely outside the previous radiation field.

Given the presence of perineural invasion and lymphovascular invasion with tumor emboli, postoperative radiotherapy (50 Gy/25 fractions) was administered to the surgical bed and regional lymph nodes using IMRT (**Figures 2A, B**). The 95% isodose line adequately encompassed the planning target volume (PTV). The clinical target volume (CTV) included the tumor bed (as identified on preoperative imaging and surgical clips), the anastomotic region, and the regional lymph node stations corresponding to the primary tumor location, including the paraesophageal, subcarinal, and paracardial lymph node regions. The planning target volume (PTV) was generated by adding a 0.5–1.0 cm margin to the CTV. The 95% isodose line adequately encompassed the PTV. Prior to the initiation of adjuvant radiotherapy, contrast esophagography using a water-soluble iodinated contrast agent (iohexol) was performed to establish a baseline, which demonstrated a patent anastomosis with smooth mucosal contours and no evidence of tumor recurrence. Postoperative radiotherapy was well tolerated. The patient experienced Grade 1 esophagitis and Grade 1 fatigue, which resolved within four weeks of treatment completion. No Grade ≥3 acute toxicities were observed. Throughout the course of adjuvant radiotherapy, serial contrast esophagography was repeated every 7–10 days to monitor for anastomotic leakage, and all examinations consistently confirmed unremarkable postoperative findings.

### 2.2 Second primary: esophageal SCC (2024)

The patient was under a structured postoperative surveillance protocol, which included periodic clinical follow-up, chest CT scans, and annual endoscopic examinations. In April 2024, sixteen months after completion of adjuvant radiotherapy, the patient reported new-onset acid reflux symptoms—likely attributable to gastric conduit reconstruction rather than the cervical lesion itself. This symptomatic presentation prompted an earlier endoscopic evaluation than originally scheduled, during which a 1.8-cm nodular lesion was incidentally detected in the cervical esophagus, located 5 cm proximal to the previous anastomotic site (**Figure 1C**). Histopathology confirmed poorly differentiated SCC. Immunohistochemical staining demonstrated P-CK(+), P40 (diffuse+), CK5/6(+), and Ki-67 (30%+), findings distinct from those of the initial ACC (**Table 1**, **Figure 1D**). Notably, contrast esophagography performed at this time did not reveal any discernible filling defect in the cervical esophagus; the lesion was instead identified by endoscopic ultrasound, which demonstrated tumor invasion into the muscularis propria.

**Table 1:** Comparative features of the two primary esophageal cancers.

Characteristic	First primary (2023)	Second primary (2024)
Location	Mid-esophagus	Cervical esophagus
Histology	Adenoid cystic carcinoma	Poorly differentiated SCC
Key IHC Markers	CD31+, P40(focal)	P40(diffuse), CK5/6+
Ki-67 Index	80%	30%
Treatment	Surgery + adjuvant RT	Definitive CRT

#### 2.2.1 Staging workup

Contrast-enhanced CT (neck/chest/abdomen): No distant metastasis.

Endoscopic ultrasound: Tumor invasion into muscularis propria (T2), left paratracheal lymph node (1.1 cm, N1).

Stage: cT2N1M0.

#### 2.2.2 Treatment

Definitive chemoradiotherapy was administered between May and July 2024.

Radiotherapy (**Figures 2C, D**):

PGTV (gross tumor + metastatic node): 60 Gy/28 fractions.

PTV (PGTV + 0.5 cm margin): 54 Gy/28 fractions.

#### 2.2.3 Chemotherapy

Concurrent S-1 (40 mg/m^2^ twice daily), an oral fluoropyrimidine consisting of tegafur, gimeracil, and oteracil potassium, was selected in consideration of the patient’s prior cumulative toxicity from esophagectomy and adjuvant radiotherapy, as well as its favorable tolerability profile and demonstrated non-inferiority as definitive chemoradiotherapy for esophageal squamous cell carcinoma in East Asian populations (22, 23). Definitive chemoradiotherapy was completed as planned without interruption. The patient developed Grade 2 radiation esophagitis and Grade 1 nausea, both of which were successfully managed with supportive care. Throughout the definitive chemoradiotherapy course, serial contrast esophagography was performed every 7–10 days, documenting progressive regression of the cervical lesion. A follow-up endoscopic examination performed three months after treatment completion demonstrated complete clinical remission of the cervical esophageal lesion. At the most recent follow-up in March 2026, the patient remained disease-free, with no evidence of recurrence of either malignancy on surveillance imaging, including contrast-enhanced CT of the neck, chest, and abdomen.

## 3 Discussion

### 3.1 Treatment decision-making for primary esophageal ACC

Esophageal ACC is an exceptionally rare malignancy, and current treatment approaches are primarily extrapolated from clinical experience with ACC at other anatomical sites, emphasizing radical local therapy as the principal strategy (1, 2). For resectable esophageal ACC, surgical excision remains the cornerstone of management, typically involving thoracoscopic-assisted radical esophagectomy with systematic lymph node dissection (4). Postoperative adjuvant radiotherapy is commonly applied in patients presenting with high-risk pathological features, such as perineural invasion or narrow surgical margins, to reduce the likelihood of local recurrence (24). Additionally, endoscopic submucosal dissection has been successfully performed in carefully selected early-stage cases in recent years (1, 25). It is also noteworthy that ACC originating from other organs, including the salivary, lacrimal, and mammary glands, similarly prioritizes surgical resection, though treatment parameters are adapted according to anatomical complexity and tumor invasiveness (26, 27).

Following radical surgery, and in accordance with therapeutic principles established for esophageal SCC, postoperative radiotherapy was administered to the surgical bed and regional lymph nodes owing to the presence of definitive high-risk features in this patient, including perineural and lymphovascular invasion with tumor emboli. Given the extreme rarity of esophageal ACC, standardized guidelines for postoperative radiotherapy field design are lacking. In the present case, the decision to encompass the surgical bed and regional lymph nodes was guided by the presence of these high-risk pathological features, which are associated with an elevated risk of locoregional recurrence. The clinical target volume was defined conservatively to include the tumor bed and the immediately draining lymphatic stations, based on principles extrapolated from esophageal SCC management. Whether this approach is optimal for esophageal ACC remains to be elucidated through further case accumulation and molecular characterization. Subsequent surveillance demonstrated no recurrence of the primary ACC, confirming both the efficacy and clinical justification of combining surgery with adjuvant radiotherapy for this rare pathological subtype.

### 3.2 Management of dual primary esophageal carcinomas

Dual primary esophageal carcinomas remain uncommon in clinical practice, necessitating individualized therapeutic planning that accounts for tumor stage, histological subtype, and overall patient status (28). Management strategies are multifaceted and may include surgical resection, systemic chemotherapy, definitive chemoradiotherapy, endoscopic interventions, or targeted therapy (18, 20, 29–31). In cases of metachronous dual primary cancers, subsequent therapeutic decisions must consider cumulative toxicity from prior interventions, particularly radiotherapy and chemotherapy, while prioritizing curative control of the newly developed lesion (17, 19).

### 3.3 Pathogenesis of multiple primary malignancies

The underlying pathogenesis of dual primary carcinomas of distinct histological types remains incompletely understood. While field cancerization—characterized by widespread genetic alterations throughout the aerodigestive epithelium following prolonged carcinogen exposure—is a well-established mechanism for the development of multiple esophageal squamous cell carcinomas, the etiology of adenoid cystic carcinoma is less clearly defined and has not been definitively linked to traditional field cancerization processes. In the present case, the substantial smoking history likely contributed to the development of the metachronous SCC, but the role of such exposures in the genesis of the initial ACC remains speculative. Therefore, the sequential occurrence of ACC and SCC in this patient more accurately illustrates the phenomenon of multiple primary malignancies rather than serving as definitive evidence for a unified field cancerization process. The emergence of the second primary SCC beyond the previously irradiated area allows radiation-induced carcinogenesis to be reasonably excluded (7, 9–12). This interpretation is supported by two key observations: first, radiation-associated malignancies typically develop within regions exposed to high-dose irradiation, whereas the new lesion in this patient was clearly located outside the 95% isodose contour; and second, radiation-induced carcinogenesis generally exhibits a latency period exceeding 10 years, in contrast to the short 16-month interval observed in this case. Consequently, these findings support the independent origins of the two neoplasms. The success of this case underscores the value of a multidisciplinary, sequential treatment strategy integrating radical local therapies for two histologically distinct esophageal malignancies. The initial ACC achieved durable control through surgery followed by adjuvant radiotherapy, while the subsequent SCC responded completely to definitive chemoradiotherapy. This outcome demonstrates that curative-intent therapy remains viable for metachronous malignancies, even in patients with a prior history of thoracic irradiation, when managed through careful multidisciplinary coordination and precise dose planning (13–16).

### 3.4 Implications and limitations

Nevertheless, several limitations must be acknowledged. Although the patient achieved favorable short-term results, the follow-up duration remains relatively short, and long-term survival, recurrence risk, and quality of life warrant continued observation. Furthermore, due to limited resources, comprehensive molecular profiling was not performed. As a result, the potential clonal relationships between the two histologically distinct tumors and the molecular pathways underlying their independent origins remain undetermined. Future investigations should emphasize detailed molecular characterization of such rare dual primary cases to elucidate pathogenic mechanisms and refine individualized therapeutic strategies.

## 4 Conclusion

This report presents, to our knowledge, the first documented case of successfully managed metachronous dual primary esophageal carcinomas consisting of the exceptionally rare ACC followed by SCC in which radiotherapy was employed as a component of curative-intent treatment for both malignancies. The favorable outcome was achieved through a precisely coordinated, sequential multimodal strategy—surgical resection with adjuvant radiotherapy for the initial ACC and definitive chemoradiotherapy for the subsequent SCC. The clear spatial and temporal separation of the second primary SCC from the previous radiation field supports their independent origins and excludes radiation-induced carcinogenesis. This case underscores that aggressive, stage-appropriate local therapy can achieve durable, disease-free survival even in complex cases of multiple primary malignancies. Furthermore, it highlights the critical importance of maintaining vigilant endoscopic surveillance in patients treated for esophageal ACC, as early detection of metachronous lesions enables curative-intent intervention.

## Data Availability

The original contributions presented in the study are included in the article/supplementary material. Further inquiries can be directed to the corresponding authors.
